# New Thiazole Nortopsentin Analogues Inhibit Bacterial Biofilm Formation

**DOI:** 10.3390/md16080274

**Published:** 2018-08-04

**Authors:** Anna Carbone, Barbara Parrino, Maria Grazia Cusimano, Virginia Spanò, Alessandra Montalbano, Paola Barraja, Domenico Schillaci, Girolamo Cirrincione, Patrizia Diana, Stella Cascioferro

**Affiliations:** Department of Biological, Chemical and Pharmaceutical Sciences and Technologies (STEBICEF), University of Palermo, Via Archirafi 32, 90100 Palermo, Italy; anna.carbone@unipa.it (A.C.); barbara.parrino@unipa.it (B.P.); mariagrazia.cusimano@unipa.it (M.G.C.); virginia.spano@unipa.it (V.S.); alessandra.montalbano@unipa.it (A.M.); paola.barraja@unipa.it (P.B.); domenico.schillaci@unipa.it (D.S.); girolamo.cirrincione@unipa.it (G.C.); stellamaria.cascioferro@unipa.it (S.C.);

**Keywords:** marine alkaloids, nortopsentin analogues, thiazole derivatives, anti-virulence agents, antibiofilm agents

## Abstract

New thiazole nortopsentin analogues were conveniently synthesized and evaluated for their activity as inhibitors of biofilm formation of relevant Gram-positive and Gram-negative pathogens. All compounds were able to interfere with the first step of biofilm formation in a dose-dependent manner, showing a selectivity against the staphylococcal strains. The most active derivatives elicited IC_50_ values against *Staphylococcus aureus* ATCC 25923, ranging from 0.40–2.03 µM. The new compounds showed a typical anti-virulence profile, being able to inhibit the biofilm formation without affecting the microbial growth in the planktonic form.

## 1. Introduction

Antibiotic resistance has become a severe global health risk, and this is partly due to excessive use of antimicrobial agents. It is estimated that in the United States alone, more than 2 million people per year are infected by antibiotic-resistant pathogens. Drug-resistant infections lead to about 23,000 deaths in the United States and 25,000 in Europe every year, and the number is higher in developing countries [1,2].

About 60–80% of bacterial infections are biofilm-mediated [3]. Biofilms are surface-attached microbial communities encased within an extracellular self-synthesized matrix able to grow both on different biotic or abiotic surfaces as indwelling devices. The biofilm architecture allows the microbes to survive in adverse conditions and it makes bacterial cells 1000 times more resistant to conventional antibiotics than the planktonic form of life of the same strains [4]. At cellular level, bacteria can develop three major mechanisms to make conventional antibiotic treatments ineffective: (i) enzymatic resistance, such as the production of β-lactamases; (ii) structural changes in the antibiotic target; and (iii) modifications in cell permeability, for example by efflux pumps. Bacterial cells in biofilm, besides these resistance mechanisms, have additional defenses because they are protected by the matrix, which prevents the entry of antibiotics, and the deepest layers undergo a metabolic inactivation that lead to the formation of dormant persister cells naturally resistant to most antibiotics.

Biofilms are responsible for a wide range of serious chronic diseases such as endocarditis, otitis media, periodontitis, prostatitis, and urinary infections. Several bacteria, including Gram-positive pathogens such as *Staphylococcus aureus*, *Streptococcus pneumoniae* and Gram-negative pathogens such as *Escherichia coli*, and *Pseudomonas aeruginosa* are often the causes of biofilm-associated infections, which are extremely challenging to treat [5].

Despite many efforts having been made in the last few years and several compounds being reported as antibiofilm agents [6,7,8,9], no derivative has reached clinical use. Therefore, there is an urgent need for the development of new therapeutic strategies effective in inhibiting biofilm formation or in dispersing preformed biofilm.

The marine environment is an important source of secondary metabolites endowed with antimicrobial activity. In particular, marine sponges are a rich source of antibacterial compounds with different mode of action. Dihydrosventrin and sventrin, bromopyrrole alkaloids, isolated from marine sponges, are biofilm inhibitors at 51 and 74 µM against *P. aeruginosa* [10]. The 2-aminoimidazole oroidin, a marine alkaloid, isolated from the marine sponge *Agelas conifer*, and its analogues are studied for antibiofilm activity [11]. Sortase A (SrtA), a transpeptidase involved in the anchoring of surface proteins to the Gram-positive bacterial cell wall, plays a key role in bacterial adhesion, immune evasion and biofilm formation [12,13]. 1*H*-Benzo[de][1,6]-naphthyridine alkaloid isoaaptamine, isolated from the marine sponge *Aaptos aaptos* [14], was reported to be a potent inhibitor of SrtA (IC_50_ value of 3.7 µM). Topsentins and hamacanthins are representative examples of marine-derived compounds displaying SrtA inhibitory activity, in particular deoxytopsentin and 6′′-debromohamacanthin A, bis(indole)alkaloids isolated from the marine sponge *Spongosorites* sp., showed IC_50_ values of 15.67 µM and 34.04 µM, respectively [15]. 

In the framework of our research on polycyclic nitrogen systems, [16,17,18,19,20,21,22,23,24,25,26,27,28,29,30,31,32,33] particularly referring to nortopsentin alkaloid analogues [34,35,36,37,38,39], herein we report the synthesis of the new series of thiazoles **1** (Table 1) and their evaluation as antibiofilm agents. In this series of nortopsentin analogues, the imidazole core of the natural product is replaced by the thiazole ring and one of the indole units is replaced by a 7-aza-indole moiety decorated with an ethanamine chain bound to the imine nitrogen. The evaluation as antibiofilm agents was performed on both the new thiazoles **1** and their *N*-2-methoxyethyl analogues **2** (Table 2), previously reported by us as antitumor agents [39], against three bacterial reference strains: *S. aureus* ATCC 25923, *S. aureus* ATCC 6538 and *P. aeuruginosa* ATCC 15442.

## 2. Results and Discussion

### 2.1. Chemistry

**Scheme 1:**
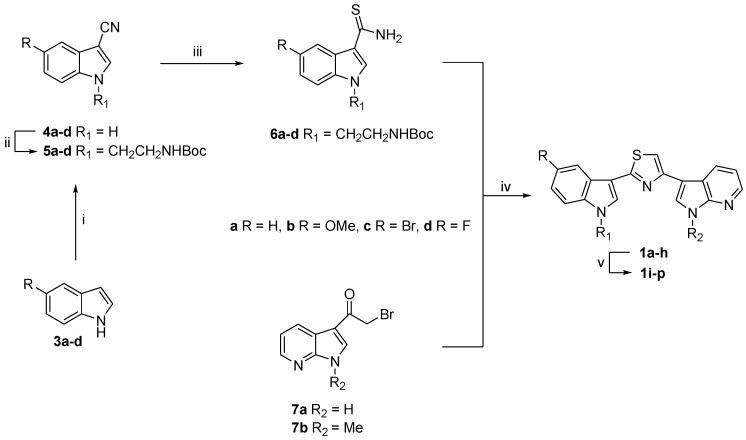
Synthesis of new thiazoles **1i**–**p**. Reagents: (**i**) (**a**) CSI, MeCN, 0 °C, 2 h; (**b**) DMF, 0 °C, 2 h, 90–98%; (**ii**) (**a**), NaH, DMF, 0 °C-rt, 30 min; (**b**) BrCH_2_CH_2_NHBoc, 60 °C, 24 h, 61–82%; (**iii**) P_4_S_10_, EtOH, rt, 1 h; then reflux, 24 h, 60–72%; (**iv**) EtOH, reflux, 30 min, 61–87%; (**v**) (**a**) TFA, DCM, reflux, 24 h; (**b**) aq NaHCO_3_, 60–91%.

Thiazoles of type **1** were conveniently prepared by Hantzsch reaction between thioamides **6a**–**d** and α-bromoacetyl compounds **7a**,**b** (Scheme 1). Indole-3-carbothioamides **6a**–**d** were obtained from the corresponding *tert*-butyl [2-(3-cyano-1*H*-indol-1-yl)ethyl]carbamates **5a**–**d**, easily prepared (61–82%) by reaction of the corresponding carbonitriles **4a**–**d** with *tert*-butyl (2-bromoethyl)carbamate using *N*,*N*-dimethylformamide (DMF) as solvent and sodium hydride as base. Carbonitriles **4a**–**d** were synthesized (90–98%) from the commercially available indoles **3a**–**d**, which were reacted with chlorosulfonyl isocyanate (CSI) in acetonitrile, followed by the addition of *N*,*N*-dimethylformamide (DMF). The reaction of carbonitriles **5a**–**d** with phosphorus pentasulfide (P_4_S_10_), under reflux in ethanol, gave the desired thioamides **6a**–**d** in good yields (60–72%). α-Bromoacetyl compounds **7a**,**b** were obtained as previously reported by us [40]. The reaction of thioamides of type **6** with α-bromoacetyl compounds **7a**,**b**, in ethanol under reflux, gave the desired thiazoles **1a**–**h** (61–87%). Their subsequent deprotection using trifluoacetic acid (TFA) in refluxing dichoromethane (DCM) led, after neutralization, to the corresponding thiazoles **1i**–**p** (60–91%) (Table 1). 

The reaction of thioamides **6c**,**d** with the 3-bromoacetyl derivative **7a** gave very unstable compounds that were used for the next step without purification.

### 2.2. Biology

The new synthesized thiazoles **1** and their previously described *N*-2-methoxyethyl analogues of type **2** were tested against *S. aureus* ATCC 25923, *S. aureus* ATCC 6538 and *P. aeuruginosa* ATCC 15442 to evaluate their ability to inhibit biofilm formation and microbial growth.

All new compounds were preliminarily assayed against the planktonic form and they did not affect the microbial growth, showing Minimum Inhibitory Concentrations (MIC) values greater than 100 µg/mL. 

Inhibition of biofilm formation of reference staphylococcal strains and *P. aeruginosa* was evaluated at sub-MIC concentrations, and IC_50_ values were determined and reported in Table 3. All tested thiazole derivatives, except **2l** and **2o,** were active as inhibitors of staphylococcal biofilm formation of both reference strains. Compounds **1p**, **2i**, **2j**, and **2n** were the most active against *S. aureus* ATCC 25923, eliciting IC_50_ values of 1.2 µM (0.5 µg/mL), 1.7 µM (0.79 µg/mL), 2.0 µM (0.95 µg/mL) and 0.4 µM (0.2 µg/mL), respectively.

Compounds **1a** and **2r** showed the best selectivity against staphylococcal biofilm formation as they showed IC_50_ values against *S. aureus* ATCC 25923 of 8.4 µM (2.9 µg/mL) and 3.7 µM (1.8 µg/mL), respectively, without affecting *P. aeruginosa* biofilm formation. The thiazole derivatives of the series **1** were more active than those of the series **2** in inhibiting Gram-negative biofilm formation. The highest potency against *P. aeruginosa* was observed for **1p** whose IC_50_ value was 9.9 µM (3.9 µg/mL). In the series **2**, only **2i** was able to inhibit pseudomonal biofilm formation, showing an IC_50_ value of 9.7 µM (4.4 µg/mL).

All the compounds were also tested, at the screening concentration of 100 µg/mL, for their dispersal activity against the preformed staphylococcal biofilm, but none were able to disrupt biofilm architecture.

**Figure 1:**
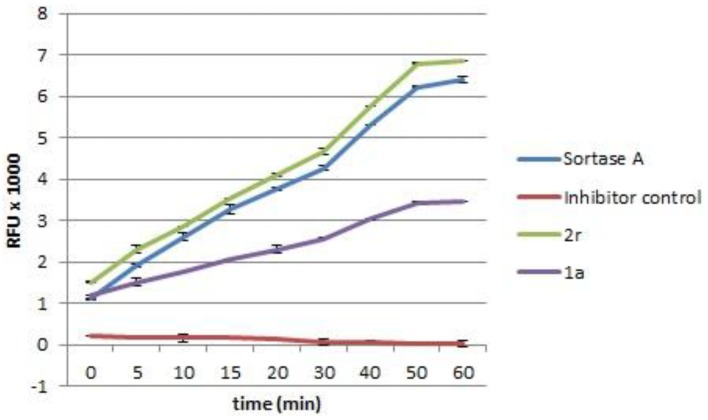
Inhibition of sortase activity by sortase inhibitor 4-(hydroxymercuri)benzoic acid (red) and **1a** (purple) and **2r** (green) and the negative control (blue) as measured with SensoLyte^®^ 520 Sortase A assay kit.

Considering that most of the synthesized compounds were selective towards Gram-positive biofilms, we selected the most potent inhibitors of staphylococcal biofilm formation, **1a** and **2r**, for further studies to elucidate the possible mechanism of action. First, we hypothesized a possible interference with the transpeptidase activity of the enzyme SrtA. A screening concentration of 100 µM **1a** showed an inhibition of 47.8%, whereas **2r**, despite its higher potency against the biofilm formation, was inactive (Figure 1).

Even if **1a** was able to inhibit SrtA activity, further studies on the anti-adhesion mechanism of action are needed. However, the new compounds showed an interesting anti-virulence behavior being capable of interfering with the biofilm formation process, which represents one of the most relevant virulence factors of many pathogens, without affecting microbial viability and imposing a low selective pressure for the evolution of antibiotic resistance mechanisms.

## 3. Materials and Methods

### 3.1. Chemistry

#### 3.1.1. General

All melting point were taken on a Büchi-Tottoly capillary apparatus (Büchi, Cornaredo, Italy) and are uncorrected. IR spectra were determined in bromoform with a Shimadzu FT/IR 8400S spectrophotometer (Shimadzu Corporation, Milan, Italy). ^1^H and ^13^C NMR spectra were measured at 200 and 50.0 MHz, respectively, in DMSO-*d_6_* solution, using a Bruker Avance II series 200 MHz spectrometer (Bruker, Milan, Italy). Column chromatography was performed with Merk silica gel 230–400 mesh ASTM (Sigma Aldrich, Milan, Italy) or with Büchi Sepacor chromatography module (prepacked cartridge system) (Büchi, Cornaredo, Italy). Elemental analyses (C, H, N) were within ±0.4% of theoretical values and were performed with a VARIO EL III elemental analyzer (Elementar, Langenselbold, Germany). Purity of all the tested compounds was greater than 95%, determined by HPLC (Agilent 1100 Series) (Agilent Technologies, Milan, Italy).

#### 3.1.2. General Procedure for the Synthesis of 1*H*-Indole-3-carbonitriles (**4a**–**d**)

To a solution of the appropriate indoles **3a**–**d** (6.8 mmol) in anhydrous acetonitrile (6.0 mL), chlorosulfonyl isocyanate (CSI) (0.63 mL, 7.25 mmol) was added dropwise at 0 °C and the reaction mixture was stirred at 0 °C for 2 h. Anhydrous dimethylformamide (DMF) (1.3 mL, 170.0 mmol) was added dropwise and the mixture was stirred at 0 °C for 2 h. The mixture was poured into ice-water and the obtained precipitate was filtered off, dried (anhydrous Na_2_SO_4_) and purified by column chromatography using petroleum ether/ethyl acetate (40/60) (for **4b**–**d**) or ethyl acetate (for **4a**) as eluent.

1*H*-In.dole-3-carbonitrile (**4a**)

White solid; yield: 96%; mp: 181 °C; spectroscopic data are in accordance with those reported in literature [41]. 

5-Met.hoxy-1*H*-indole-3-carbonitrile (**4b**)

White solid; yield: 90%; mp: 157 °C; spectroscopic data in accordance with those reported in literature [41]. 

5-Bro.mo-1*H*-indole-3-carbonitrile (**4c**)

White solid; yield: 91%; mp: 193 °C; IR cm^−1^: 2219 (CN), 3440 (NH); ^1^H NMR (200 MHz, DMSO-*d_6_*) δ: 7.42 (dd, 1H, *J* = 8.7, 1.8 Hz, H-6), 7.54 (d, 1H, *J* = 8.7 Hz, H-7), 7.81 (d, 1H, *J* = 1.8 Hz, H-4), 8.33 (s, 1H, H-2), 12.42 (bs, 1H, NH); ^13^C NMR (50 MHz, DMSO-*d_6_*) δ: 83.9 (C), 114.4 (C), 115.0 (CH), 115.7 (C), 120.7 (CH), 126.1 (CH), 128.4 (C), 134.0 (CN), 135.9 (CH). Anal. Calcd. for: C_9_H_5_BrN_2_: C, 48.90; H, 2.28; N, 12.67. Found: C, 48.74; H, 2.44; N, 12.93.

5-Flu.oro-1*H*-indole-3-carbonitrile (**4d**)

White solid; yield: 98%; mp: 182 °C; spectroscopic data in accordance with those reported in literature. [41]

#### 3.1.3. General Procedure for the Synthesis of *Tert*-Butyl [2-(3-cyano-1*H*-indol-1-yl)ethyl]carbamates (**5a**–**d**)

To a cold solution of the appropriate indoles **4a**–**d** (4.2 mmol) in anhydrous DMF (7.8 mL) NaH (60% suspension in mineral oil, 6.3 mmol, 0.25 g) was added. After 30 min stirring at room temperature, *tert*-butyl (2-bromoethyl)carbamate [42] (6.3 mmol, 1.4 g) was added. The reaction mixture was heated at 60 °C for 24 h. After cooling, the mixture was poured into ice-water and extracted with ethyl acetate (3 × 20 mL). The organic phases were dried (anhydrous Na_2_SO_4_) and evaporated under reduced pressure. The residue was purified by column chromatography using petroleum ether/ethyl acetate (70/30) (for **5b**–**d**) or petroleum ether/ethyl acetate (50/50) (for **5a**) as eluent.

*Tert*-Butyl [2-(3-cyano-1*H*-indol-1-yl)ethyl]carbamate (**5a**)

White solid; yield: 80%; mp: 143 °C; IR cm^−1^: 3335 (NH), 2220 (CN), 1706 (CO); ^1^H NMR (200 MHz, DMSO-*d_6_*) δ: 1.30 (s, 9H, 3 × CH_3_), 3.28–3.36 (m, 2H, CH_2_), 4.30 (t, 2H, *J* = 5.8 Hz, CH_2_), 6.98 (t, 1H, *J* = 5.6 Hz, NH), 7.23–7.38 (m, 2H, H-5 and H-6), 7.63–7.67 (m, 2H, H-4 and H-7), 8.21 (s, 1H, H-2); ^13^C NMR (50 MHz, DMSO-*d_6_*) δ: 28.0 (3 × CH_3_), 40.0 (CH_2_), 45.9 (CH_2_), 77.8 (C), 83.6 (C), 111.4 (CH), 116.1 (C), 118.6 (CH), 121.8 (CH), 123.3 (CH), 127.1 (C), 135.6 (CN), 137.0 (CH), 155.5 (CO). Anal. Calcd. for: C_16_H_19_N_3_O_2_: C, 67.35; H, 6.71; N, 14.73. Found: C, 67.28; H, 6.89; N, 14.90.

*Tert*-Butyl [2-(5-methoxy-3-cyano-1*H*-indol-1-yl)ethyl]carbamate (**5b**)

White solid; yield: 61%; mp: 149 °C; IR cm^−C1^: 3382 (NH), 2212 (CN), 1703 (CO); ^1^H NMR (200 MHz, DMSO-*d_6_*) δ: 1.31 (s, 9H, 3 × CH_3_), 3.26–3.36 (m, 2H, CH_2_), 3.82 (s, 3H, CH_3_), 4.25 (t, 2H, *J* = 5.6 Hz, CH_2_), 6.92–7.00 (m, 2H, H-6 and NH), 7.08 (d, 1H, *J* = 2.2 Hz, H-4), 7.55 (d, 1H, *J* = 9.0 Hz, H-7), 8.12 (s, 1H, H-2); ^13^C NMR (50 MHz, DMSO-*d_6_*) δ: 28.0 (3 × CH_3_), 40.0 (CH_2_), 46.0 (CH_2_), 55.4 (CH_3_), 77.8 (C), 83.4 (C), 100.0 (CH), 112.3 (CH), 113.6 (CH), 116.3 (C), 127.9 (C), 130.4 (C), 136.8 (CH), 155.4 (CN), 155.5 (CO). Anal. Calcd. for: C_17_H_21_N_3_O_3_: C, 64.74; H, 6.71; N, 13.32. Found: C, 64.63; H, 6.95; N, 13.57.

*Tert*-Butyl [2-(5-bromo-3-cyano-1*H*-indol-1-yl)ethyl]carbamate (**5c**) 

White solid; yield: 82%; mp: 183 °C; IR cm^−1^: 3356 (NH), 2221 (CN), 1684 (CO); ^1^H NMR (200 MHz, DMSO-*d_6_*) δ: 1.26 (s, 9H, 3 × CH_3_), 3.26–3.40 (m, 2H, CH_2_), 4.29 (t, 2H, *J* = 5.6 Hz, CH_2_), 6.96 (t, 1H, *J* = 5.8 Hz, NH), 7.48 (dd, 1H, *J* = 8.8, 2.0 Hz, H-6), 7.64 (d, 1H, *J* = 8.8 Hz, H-7), 7.80 (d, 1H, *J* = 2.0 Hz, H-4), 8.26 (s, 1H, H-2); ^13^C NMR (50 MHz, DMSO-*d_6_*) δ: 27.4 (3 × CH_3_), 39.9 (CH_2_), 46.3 (CH_2_), 77.8 (C), 83.3 (C), 113.6 (CH), 114.6 (C), 115.4 (C), 120.9 (CH), 126.0 (CH), 128.7 (C), 134.5 (CO), 138.4 (CH), 155.4 (CN)**.** Anal. Calcd. for: C_16_H_18_BrN_3_O_2_: C, 52.76; H, 4.98; N, 11.54. Found: C, 52.61; H, 5.24; N, 11.67.

*Tert*-Butyl [2-(5-fluoro-3-cyano-1*H*-indol-1-yl)ethyl]carbamate (**5d**)

White solid; yield: 65%; mp: 182 °C; IR cm^−1^: 3357 (NH), 2221 (CN), 1701 (CO); ^1^H NMR (200 MHz, DMSO-*d_6_*) δ: 1.28 (s, 9H, 3 × CH_3_), 3.30–3.35 (m, 2H, CH_2_), 4.29 (t, 2H, *J* = 5.1 Hz, CH_2_), 6.97 (t, 1H, *J* = 6.0 Hz, NH), 7.22 (td, 1H, *J* = 11.4, 9.2, 2.3 Hz, H-6), 7.43 (dd, 1H, *J* = 11.4, 2.3 Hz, H-4), 7.69 (dd, 1H, *J* = 9.2, 4.2 Hz, H-7), 8.27 (s, 1H, H-2); ^13^C NMR (50 MHz, DMSO-*d_6_*) δ: 77.8 (C), 83.8 (C, *J_C7a-F_* = 4.2 Hz), 103.9 (CH, *J_C6-F_* = 24.6 Hz), 111.7 (CH, *J_C4-F_* = 26.2 Hz), 112.8 (C), 113.0 (CH, *J_C7-F_* = 9.5 Hz), 115.6 (C), 127.6 (C, *J_C3a-F_* = 11.0 Hz), 132.3 (C), 138.6 (CH), 158.5 (C, *J_C5-F_* = 238 Hz). Anal. Calcd. for: C_16_H_18_FN_3_O_2_: C, 63.35; H, 5.98; N, 13.85. Found: C, 63.18; H, 6.14; N, 13.67.

#### 3.1.4. General Procedure for the Synthesis of *Tert*-Butyl [2-(3-carbamothioyl-1*H*-indol-1-yl)ethyl]carbamate (**6a**–**d**)

A solution of phosphorus pentasulfide (P_4_S_10_) (0.73 g, 1.64 mmol) in anhydrous ethanol (2.0 mL) was stirred at room temperature for 1 h. The appropriate indole carbonitriles **5a**–**d** (0.3 g, 0.82 mmol) was added and the reaction mixture was heated under reflux for 24 h. Water (20 mL) was added and the reaction mixture was extracted with ethyl acetate (3 × 20 mL). The organic phases were dried (anhydrous Na_2_SO_4_) and evaporated under reduced pressure. The residue was purified by column chromatography using dichloromethane/ethyl acetate (70:30) as eluent.

*Tert*-Butyl [2-(3-carbamothioyl-1*H*-indol-1-yl)ethyl]carbamate (**6a**)

Yellow solid; yield: 60%; mp: 162 °C; IR cm^−1^: 3389 (NH), 3375, 3448 (NH_2_), 1707 (CO), 1595 (CS); ^1^H NMR (200 MHz, DMSO-*d_6_*) δ: 1.35 (s, 9H, 3 × CH_3_), 3.30–3.36 (m, 2H, CH_2_), 4.26 (t, 2H, *J* = 5.9 Hz, CH_2_), 7.02 (t, 1H, *J* = 5.4 Hz, NH), 7.14–7.27 (m, 2H, H-5 and H-6), 7.50–7.55 (m, 1H, H-7), 8.09 (s, 1H, H-2), 8.57 (d, 1H, *J* = 6.8 Hz, H-4), 8.78 (bs, 1H, SH), 9.00 (bs, 1H, NH); ^13^C NMR (50 MHz, DMSO-*d_6_*) δ: 27.4 (3 × CH_3_), 40.1 (CH_2_), 45.3 (CH_2_), 77.9 (C), 110.3 (CH), 115.8 (C), 121.0 (CH), 121.8 (CH), 122.1 (CH), 126.0 (C), 131.9 (CH), 136.5 (C), 155.6 (CO), 193.1 (CS). Anal. Calcd. for: C_16_H_21_N_3_O_2_S: C, 60.16; H, 6.63; N, 13.16. Found: C, 60.02; H, 6.89; N, 13.40.

*Tert*-Butyl [2-(3-carbamothioyl-5-methoxy-1*H*-indol-1-yl)ethyl]carbamate (**6b**)

Yellow solid; yield: 72%; mp: 172 °C; IR cm^−1^: 3382 (NH), 3265, 3178 (NH_2_), 1688 (CO), 1525 (CS); ^1^H NMR (200 MHz, DMSO-*d_6_*) δ: 1.35 (s, 9H, 3 × CH_3_), 3.25–3.33 (m, 2H, CH_2_), 3.79 (s, 3H, CH_3_), 4.22 (t, 2H, *J* = 5.8 Hz, CH_2_), 6.87 (dd, 1H, *J* = 8.9, 2.5 Hz, H-6), 7.01 (t, 1H, *J* = 5.5 Hz, NH), 7.42 (d, 1H, *J* = 8.9 Hz, H-7), 8.06 (s, 1H, H-2), 8.17 (d, 1H, *J* = 2.5 Hz, H-4), 8.73 (s, 1H, SH), 8.93 (bs, 1H, NH); ^13^C NMR (50 MHz, DMSO-*d_6_*) δ: 28.1 (3 × CH_3_), 40.3 (CH_2_), 45.5 (CH_2_), 55.3 (CH_3_), 77.9 (C), 103.9 (CH), 111.1 (CH), 111.8 (CH), 115.2 (C), 126.7 (C), 131.9 (C), 132.2 (CH), 154.9 (C), 155.6 (CO), 192.9 (CS). Anal. Calcd. for: C_17_H_23_N_3_O_3_S: C, 58.43; H, 6.63; N, 12.02. Found: C, 58.19; H, 6.37; N, 11.75.

*Tert*-Butyl [2-(3-carbamothioyl-5-bromo-1*H*-indol-1-yl)ethyl]carbamate (**6c**) 

Yellow solid; yield: 72%; mp: 172 °C; IR cm^−1^: 3278 (NH), 3402, 3371 (NH_2_), 1684 (CO), 1533 (CS); ^1^H NMR (200 MHz, DMSO-*d_6_*) δ: 1.32 (s, 9H, 3 × CH_3_), 3.28-3.37 (m, 2H, CH_2_), 4.25 (t, 2H, *J* = 5.5 Hz, CH_2_), 7.01 (t, 1H, *J* = 5.5 Hz, NH), 7.36 (dd, 1H, *J* = 8.7, 1.9 Hz, H-6), 7.52 (d, 1H, *J* = 8.7 Hz, H-7), 8.13 (s, 1H, H-2), 8.87 (d, 1H, *J* = 1.9 Hz, H-4), 8.91 (s, 1H, SH), 9.08 (bs, 1H, NH); ^13^C NMR (50 MHz, DMSO-*d_6_*) δ: 28.1 (3 × CH_3_), 40.0 (CH_2_), 45.7 (CH_2_), 77.9 (C), 112.5 (CH), 114.0 (C), 115.9 (C), 124.1 (CH), 124.6 (CH), 128.1 (C), 132.3 (CH), 135.7 (C), 155.6 (CO), 192.5 (CS). Anal. Calcd. for: C_16_H_20_BrN_3_O_2_S: C, 48.25; H, 5.06; N, 10.55. Found: C, 48.13; H, 4.95; N, 10.68.

*Tert*-Butyl [2-(3-carbamothioyl-5-fluoro-1*H*-indol-1-yl)ethyl]carbamate (**6d**) 

Yellow solid; yield: 60%; mp: 166 °C; IR cm^−1^: 3374 (NH), 3278, 3182 (NH_2_), 1686 (CO), 1526 (CS);^1^H NMR (200 MHz, DMSO-*d_6_*) δ: 1.33 (s, 9H, 3 × CH_3_), 3.29–3.37 (m, 2H, CH_2_), 4.26 (t, 2H, *J* = 5.8 Hz, CH_2_), 6.99-7.14 (m, 2H, H-6 and NH), 7.55 (dd, 1H, *J* = 9.0, 4.6 Hz, H-7), 8.16 (s, 1H, H-2), 8.42 (dd, 1H, *J* = 11.0, 2.5 Hz, H-4), 8.85 (bs, 1H, SH), 9.03 (bs, 1H, NH); ^13^C NMR (50 MHz, DMSO-*d_6_*) δ: 28.1 (3 × CH_3_), 40.0 (CH_2_), 45.8 (CH_2_), 77. 9 (C), 99.5 (C), 106.9 (CH, *J_C6-F_* = 25.8 Hz), 110.2 (CH, *J_C4-F_* = 26.0 Hz), 111.6 (CH, *J_C7-F_* = 10.1 Hz), 115.3 (C, *J_C7a-F_* = 4.5 Hz), 126.9 (C, *J_C3a-F_* = 11.2 Hz), 132.9 (CH), 133.6 (C), 155.6 (CO), 158.1 (C, *J_C5-F_* = 233 Hz), 192.6 (CS). Anal. Calcd. for: C_16_H_20_FN_3_O_2_S: C, 56.95; H, 5.97; N, 12.45. Found: C, 56.69; H, 6.25; N, 12.21.

#### 3.1.5. General Procedure for the Synthesis of Thiazoles (**1a**–**h**)

A suspension of the proper thioamides **6a**–**d** (2 mmol) and bromoacetyl derivatives **7a**,**b** (2 mmol) in anhydrous ethanol (8 mL) was refluxed for 30 min. After cooling, the precipitate obtained, was filtered off, dried, and recrystallized from ethanol to give the desired thiazoles **1a**–**h**.

*Tert*-Butyl (2-{3-[4-(1*H*-pyrrolo[2,3-*b*]pyridin-3-yl)-1,3-thiazol-2-yl]-1*H*-indol-1-yl}ethyl)carbamate (**1a**) 

Orange solid; yield: 70%; mp: 229–230 °C; IR cm^−1^: 3348 (NH), 3090 (NH), 1684 (CO); ^1^H NMR (200 MHz, DMSO-*d_6_*) δ: 1.32 (s, 9H, 3 × CH_3_), 3.35–3.46 (m, 2H, CH_2_), 4.35 (t, 2H, *J* = 5.1 Hz, CH_2_), 7.06 (t, 1H, *J* = 5.0 Hz, NH), 7.28–7.33 (m, 2H, H-5′ and H-6′), 7.41 (dd, 1H, *J* = 7.9, 5.1 Hz, H-5′′), 7.59–7.64 (m. 1H, H-7′), 7.82 (s, 1H, H-5), 8.19 (s, 1H, H-2′), 8.23 (d, 1H, *J* = 2.2 Hz, H-2′′), 8.29-8.34 (m, 1H, H-4′), 8.43 (d, 1H, *J* = 5.1 Hz, H-6′′), 8.89 (d, 1H, *J* = 7.9 Hz, H-4′′), 12.40 (bs, 1H, NH); ^13^C NMR (50 MHz, DMSO-*d_6_*) δ: 28.1 (3 × CH_3_), 40.2 (CH_2_), 45.3 (CH_2_), 77.8 (C), 108.5 (CH), 109.7 (C), 110.6 (CH), 111.2 (C), 116.2 (CH), 120.3 (CH), 120.6 (C), 121.1 (CH), 122.4 (CH), 124.6 (C), 126.5 (CH), 129.9 (CH), 134.3 (CH), 136.8 (C), 137.7 (CH), 142.7 (C), 148.1 (C), 155.6 (C), 162.2 (CO). Anal. Calcd. for: C_25_H_25_N_5_O_2_S: C, 65.34; H, 5.48; N, 15.24. Found: C, 65.30; H, 5.62; N, 15.45.

*Tert*-Butyl (2-{3-[4-(1-methyl-1*H*-pyrrolo[2,3-*b*]pyridin-3-yl)-1,3-thiazol-2-yl]-1*H*-indol-1-yl}ethyl)carbamate (**1b**) 

Yellow solid; yield: 68%; mp: 187 °C; IR cm^−1^: 3335 (NH), 1705 (CO); ^1^H NMR (200 MHz, DMSO-*d_6_*) δ: 1.32 (s, 9H, 3 × CH_3_), 3.36–3.43 (m, 2H, CH_2_), 3.94 (s, 1H, CH_3_), 4.35 (t, 2H, *J* = 5.0 Hz, CH_2_), 7.05 (t, 1H, *J* = 4.6 Hz, NH), 7.27–7.32 (m, 3H, H-5′, H-6′ and H-5′′), 7.59–7.63 (m. 1H, H-7′), 7.73 (s, 1H, H-5), 8.15 (s, 1H, Ar), 8.22 (s, 1H, Ar), 8.33–8.41 (m, 2H, H-4′ and H-6′′), 8.67 (dd, 1H, *J* = 8.0, 1.4 Hz, H-4′′); ^13^C NMR (50, DMSO-*d_6_*) δ: 28.1 (3 × CH_3_), 31.8 (CH_3_), 40.2 (CH_2_), 45.2 (CH_2_), 77.8 (C), 107.6 (CH), 109.5 (C), 109.7 (C), 110.6 (CH), 116.1 (CH), 118.9 (C), 120.5 (CH), 121.0 (CH), 122.4 (CH), 124.6 (C), 129.7 (CH), 129.9 (CH), 131.3 (CH), 136.7 (C), 140.3 (CH), 145.0 (C), 148.4 (C), 155.6 (C), 162.1 (CO). Anal. Calcd. for: C_26_H_27_N_5_O_2_S: C, 65.94; H, 5.75; N, 14.79. Found: C, 65.80; H, 5.71; N, 14.97. 

*Tert*-Butyl (2-{5-methoxy-3-[4-(1*H*-pyrrolo[2,3-*b*]pyridin-3-yl)-1,3-thiazol-2-yl]-1*H*-indol-1-yl}ethyl)carbamate (**1c**)

Yellow solid; yield: 87%; mp: 201 °C; IR cm^−1^: 3584 (NH), 3342 (NH), 1683 (CO); ^1^H NMR (200 MHz, DMSO-*d_6_*) δ: 1.32 (s, 9H, 3 × CH_3_), 3.34–3.39 (m, 2H, CH_2_), 3.90 (s, 3H, CH_3_), 4.31 (t, 2H, *J* = 5.7 Hz, CH_2_), 6.94 (dd, 1H, *J* = 8.9, 2.4 Hz, H-6′), 7.04 (t, 1H, *J* = 5.2 Hz, NH), 7.40 (dd, 1H, *J* = 7.9, 5.1 Hz, H-5′′), 7.52 (d, 1H, *J* = 8.9 Hz, H-7′), 7.79 (s, 1H, H-5), 7.89 (d, 1H, *J* = 2.3 Hz, H-2′′), 8.11 (s, 1H, H-2′), 8.22 (d, 1H, *J* = 2.4 Hz, H-4′), 8.45 (d, 1H, *J* = 5.1 Hz, H-6′′), 8.l9 (d, 1H, *J* = 7.9 Hz, H-4′′), 12.43 (bs, 1H, NH); ^13^C NMR (50 MHz, DMSO-*d_6_*) δ: 28.1 (3 × CH_3_), 40.2 (CH_2_), 45.4 (CH_2_), 55.2 (CH_3_), 77.8 (C), 102.0 (CH), 107.9 (CH), 109.4 (C), 111.1 (C), 111.5 (CH), 112.5 (CH), 116.0 (CH), 120.4 (C), 125.2 (C), 126.2 (CH), 130.1 (CH), 131.8 (C), 133.9 (CH), 138.2 (CH), 143.0 (C), 148.3 (C), 154.9 (C), 155.6 (C), 161.5 (CO). Anal. Calcd. for: C_26_H_27_N_5_O_3_S: C, 63.78; H, 5.56; N, 14.30. Found: C, 63.52; H, 5.50; N, 14.41. 

*Tert*-Butyl (2-{5-methoxy-3-[4-(1-methyl-1*H*-pyrrolo[2,3-*b*]pyridin-3-yl)-1,3-thiazol-2-yl]-1*H*-indol-1-yl}ethyl)carbamate (**1d**) 

Yellow solid; yield: 61%; mp: 191 °C; IR cm^−1^: 3360 (NH), 1707 (CO); ^1^H NMR (200 MHz, DMSO-*d_6_*) δ: 1.33 (s, 9H, 3 × CH_3_), 3.31–3.40 (m, 2H, CH_2_), 3.91 (s, 1H, CH_3_), 3.94 (s, 1H, CH_3_), 4.30 (t, 2H, *J* = 4.7 Hz, CH_2_), 6.95 (dd, 1H, *J* = 8.9, 2.4 Hz, H-6′), 7.04 (t, 1H, *J* = 5.5 Hz, NH), 7.28 (dd, 1H, *J* = 7.9, 4.7 Hz, H-5′′), 7.51 (d, 1H, *J* = 8.9 Hz, H-7′), 7.69 (s, 1H, H-5), 7.89 (d, 1H, *J* = 2.4 Hz, H-4′), 8.09 (s, 1H, Ar), 8.17 (s, 1H, Ar), 8.40 (dd, 1H, *J* = 4.7, 1.4 Hz, H-6′′), 8.77 (dd, 1H, *J* = 7.9, 1.4 Hz, H-4′′), ^13^C NMR (50 MHz, DMSO-*d_6_*) δ: 28.1 (3 × CH_3_), 31.2 (CH_3_), 40.2 (CH_2_), 45.5 (CH_2_), 55.1 (CH_3_), 77.8 (C), 101.5 (H), 106.7 (CH), 109.1 (C), 109.6 (C), 111.7 (CH), 112. 9 (CH), 116.0 (CH), 116.2 (CH), 118.9 (C), 124.9 (C), 129.2 (CH), 130.5 (CH), 131.8 (C), 143.0 (CH), 146.6 (C), 147.3 (C), 155.1 (C), 155.7 (C), 162.6 (CO). Anal. Calcd. for: C_27_H_29_N_5_O_3_S: C, 64.39; H, 5.80; N, 13.91. Found: C, 64.62; H, 5.65; N, 13.73. 

*Tert*-Butyl (2-{5-bromo-3-[4-(1*H*-pyrrolo[2,3-*b*]pyridin-3-yl)-1,3-thiazol-2-yl]-1*H*-indol-1-yl}ethyl)carbamate (1**e**) 

Very unstable compound, used in the next step without purification.

*Tert*-Butyl (2-{5-bromo-3-[4-(1-methyl-1*H*-pyrrolo[2,3-*b*]pyridin-3-yl)-1,3-thiazol-2-yl]-1*H*-indol-1-yl}ethyl)carbamate (**1f**) 

Orange solid; yield: 72%; mp: 203–204 °C; IR cm^−1^: 3337 (NH), 1704 (CO); ^1^H NMR (200 MHz, DMSO-*d_6_*) δ: 1.29 (s, 9H, 3 × CH3), 3.35–3.39 (m, 2H, CH_2_), 3.95 (s, 3H, CH_3_), 4.34 (t, 2H, *J* = 4.8 Hz, CH_2_), 6.99 (t, 1H, *J* = 5.7 Hz, NH), 7.30 (dd, 1H, *J* = 7.9, 4.7 Hz, H-5′′), 7.44 (dd, 1H, *J* = 8.8, 1.8 Hz, H-6′), 7.61 (d, 1H, *J* = 8.8 Hz, H-7′), 7.74 (s, 1H, H-5), 8.18 (s, 1H, Ar), 8.20 (s, 1H, Ar), 8.41 (dd, 1H, *J* = 4.7, 1.3 Hz, H-6′′), 8.51 (d, 1H, *J* = 1.8 Hz, H-4′), 8.69 (dd, 1H, *J* = 7.9, 1.7 Hz, H-4′′); ^13^C NMR (50 MHz, DMSO-*d_6_*) δ: 28.0 (3 × CH3), 31.5 (CH_3_), 40.2 (CH_2_), 45.6 (CH_2_), 77.8 (C), 99.5 (C), 107.6 (CH), 109.2 (C), 109.4 (C), 112.8 (CH), 113.6 (C), 116.1 (CH), 118.4 (C), 122.7 (CH), 124.9 (CH), 126.3 (C), 129.1 (CH), 130.5 (CH), 131.2 (CH), 135.6 (C), 141.2 (CH), 149.0 (C), 155.6 (C), 161.4 (CO). Anal. Calcd. for: C_26_H_26_BrN_5_O_2_S: C, 56.52; H, 4.74; N, 12.68. Found: C, 56.66; H, 4.92; N, 12.60.

*Tert*-Butyl (2-{5-fluoro-3-[4-(1*H*-pyrrolo[2,3-*b*]pyridin-3-yl)-1,3-thiazol-2-yl]-1*H*-indol-1-yl}ethyl)carbamate (**1g**) 

Very unstable compound, used in the next step without purification

*Tert*-Butyl (2-{5-fluoro-3-[4-(1-methyl-1*H*-pyrrolo[2,3-*b*]pyridin-3-yl)-1,3-thiazol-2-yl]-1*H*-indol-1-yl}ethyl)carbamate (**1h**)

Yellow solid; yield: 86%; mp: 193–194 °C; IR cm^−1^: 3329 (NH), 1704 (CO); ^1^H NMR (200 MHz, DMSO-*d_6_*) δ:. 1.30 (s, 9H, 3 × CH3), 3.35–3.41 (m, 2H, CH_2_), 3.96 (s, 3H, CH_3_), 4.34 (t, 2H, *J* = 4.9 Hz, CH_2_), 7.04 (t, 1H, *J* = 5.1 Hz, NH), 7.18 (td, 1H, *J* = 11.7, 9.1, 2.5 Hz, H-6′), 7.32 (dd, 1H, *J* = 7.9, 4.8 Hz, H-5′′), 7.74 (s, 1H, H-5), 7.64 (dd, 1H, *J* = 9.1, 4.6 Hz, H-7′), 8.09 (dd, 1H, *J* = 11.7, 2.5 Hz, H-4′), 8.22 (s, 1H, Ar), 8.25 (s, 1H, Ar), 8.42 (dd, 1H, *J* = 4.8, 2.0 Hz, H-6′′), 8.68 (dd, 1H, *J* = 7.9, 2.0 Hz, H-4′′); ^13^C NMR (50 MHz, DMSO-*d_6_*) δ: 28.1 (3 × CH3), 31.9 (CH_3_), 40.2 (CH_2_), 45.6 (CH_2_), 99.5 (CH), 105.5 (CH, *J_C6′-F_* = 25.0 Hz), 107.8 (CH), 109.4 (C), 109.8 (C), 110.0 (C), 110.7 (CH, *J_C4′-F_* = 26.6 Hz), 111.9 (CH, *J_C7-F_* = 9.7 Hz), 112.0 (C), 116.1 (CH), 118.5 (C), 124.9 (C, *J_C3′a-F_* = 10.2 Hz), 129.9 (CH), 131.6 (CH), 133.5 (C), 140.0 (CH), 148.7 (C), 155.8 (C), 158.0 (CH, *J_C5-F_* = 246 Hz), 161.7 (CO). Anal. Calcd. for: C_26_H_26_FN_5_O_2_S: C, 63.53; H, 5.33; N, 14.25. Found: C, 63.67; H, 5.29; N, 14.32.

#### 3.1.6. General Procedure for the Synthesis of Thiazoles (**1i**–**p**)

To a suspension of appropriate thiazoles **1a**–**h** (0.38 mmol) in DCM (5 mL) trifluoroacetic acid (0.54 mL, 7.0 mmol) was added and the mixture was heated under reflux for 24 h. After cooling, the mixture was neutralized with saturated aqueous sodium hydrogen carbonate solution and extracted with dichloromethane (3 × 20 mL). The organic phases were dried (anhydrous Na_2_SO_4_), evaporated under reduced pressure, and the residue was recrystallized with ethanol to afford the desired thiazoles **7i**–**p**.

2-{3-[4-(1*H*-Pyrrolo[2,3-*b*]pyridin-3-yl)-1,3-thiazol-2-yl]-1*H*-indol-1-yl}ethanamine (**1i**) 

Yellow solid; yield: 65%; mp: 165 °C; IR cm^−1^: 3608, 3558 (NH_2_), 3249 (NH); ^1^H NMR (200 MHz, DMSO-*d_6_*) δ: 3.34–3.37 (m, 2H, CH_2_), 4.56 (t, 2H, *J* = 5.7 Hz, CH_2_), 7.29–7.37 (m, 3H, H-5′, H-6′ and H-5′′), 7.69-7.73 (m. 1H, H-7′), 7.80 (s, 1H, H-5), 8.02 (bs, 2H, NH_2_), 8.17 (d, 1H, *J* = 2.4 Hz, H-2′′), 8.28 (s, 1H, H-2′), 8.28–8.39 (m, 2H, H-4′ and H-6′′), 8.76 (d, 1H, *J* = 7.7 Hz, H-4′′), 12.22 (bs, 1H, NH); ^13^C NMR (50 MHz, DMSO-*d_6_*) δ: 38.5 (CH_2_), 43.5 (CH_2_), 107.8 (C), 110.5 (CH), 110.6 (CH), 116. 2 (CH), 118.7 (C), 120.6 (CH), 121.4 (CH), 122.7 (CH), 124.9 (C), 125.5 (CH), 129.8 (CH), 131.1 (CH), 131.3 (C), 136.5 (C), 140.7 (CH), 146.0 (C), 149.2 (C), 161.6 (CO). Anal. Calcd. for: C_20_H_17_N_5_S: C, 66.83; H, 4.77; N, 19.48. Found: C, 66.97; H, 4.63; N, 19.65.

2-{3-[4-(1-Methyl-1*H*-pyrrolo[2,3-*b*]pyridin-3-yl)-1,3-thiazol-2-yl]-1*H*-indol-1-yl}ethanamine (**1j**) 

Yellow solid; yield: 70%; mp: 179 °C; IR cm^−1^: 3598, 3559 (NH_2_); ^1^H NMR (200 MHz, DMSO-*d_6_*) δ: 3.30–3.40 (m, 2H, CH_2_), 3.94 (s, 1H, CH_3_), 4.56 (t, 2H, *J* = 5.9 Hz, CH_2_), 7.26-7.40 (m, 3H, H-5′, H-6′ and H-5′′), 7.69-7.75 (m. 2H, H-5 and H-7′), 8.03 (bs, 2H, NH_2_), 8.22 (s, 1H, Ar), 8.27 (s, 1H, Ar), 8.38–8.43 (m, 2H, H-4′ and H-6′′), 8.67 (dd, 1H, *J* = 7.9, 1.4 Hz, H-4′′); ^13^C NMR (50 MHz, DMSO-*d_6_*) δ: 31.1 (CH_3_), 38.5 (CH_2_), 43.5 (CH_2_), 107.1 (CH), 109.0 (C), 110.6 (CH), 116.2 (CH), 117.6 (C), 120.8 (CH), 121.3 (CH), 122.7 (CH), 125.0 (C), 128.9 (CH), 129.2 (CH), 129.8 (CH), 136.5 (C), 142.4 (CH), 147.2 (C), 149.3 (C), 158.7 (C), 161.6 (CO). Anal. Calcd. for: C_21_H_19_N_5_S: C, 67.53; H, 5.13; N, 18.75. Found: C, 67.48; H, 5.10; N, 18.99.

2-{5-Methoxy-3-[4-(1*H*-pyrrolo[2,3-*b*]pyridin-3-yl)-1,3-thiazol-2-yl]-1*H*-indol-1-yl}ethanamine (**1k**)

Yellow solid; yield: 70%; mp: 220 °C; IR cm^−1^: 3609, 3557 (NH_2_), 3452 (NH); ^1^H NMR (200 MHz, DMSO-*d_6_*) δ: 3.29–3.37 (m, 2H, CH_2_), 3.91 (s, 1H, CH_3_), 4.52 (t, 2H, *J* = 5.7 Hz, CH_2_),7.00 (dd, 1H, *J* = 8.9, 2.4 Hz, H-6′), 7.30 (dd, 1H, *J* = 7.9, 4.9 Hz, H-5′′), 7.62 (d, 1H, *J* = 8.9 Hz, H-7′), 7.77 (s, 1H, H-5), 7.94 (d, 1H, *J* = 2.3 Hz, H-2′′), 8.03 (bs, 2H, NH_2_), 8.17 (d, 1H, *J* = 2.4 Hz, H-4′), 8.21 (s, 1H, H-2′), 8.38 (d, 1H, *J* = 4.9 Hz, H-6′′), 8.86 (d, 1H, *J* = 7.9 Hz, H-4′′), 12.23 (bs, 1H, NH); ^13^C NMR (50 MHz, DMSO-*d_6_*) δ: 38.5 (CH_2_), 43.6 (CH_2_), 55.2 (CH_3_), 102.2 (CH), 107.2 (CH), 110.3 (C), 110.5 (C), 111.5 (CH), 112.7 (CH), 116.0 (CH), 118.6 (C), 125.2 (CH), 125.5 (C), 130.1 (CH), 130.9 (CH), 131.5 (C), 141.0 (CH), 146.3 (C), 149.3 (C), 155.1 (C), 161.9 (C). Anal. Calcd. for: C_21_H_19_N_5_OS: C, 64.76; H, 4.92; N, 17.98. Found: C, 64.92; H, 4.83; N, 18.09.

2-{5-Methoxy-3-[4-(1-methyl-1*H*-pyrrolo[2,3-*b*]pyridin-3-yl)-1,3-thiazol-2-yl]-1*H*-indol-1-yl}ethanamine (**1l**)

Yellow solid; yield: 75%; mp: 232 °C; IR cm^−1^: 3608, 3558 (NH_2_); ^1^H NMR (200 MHz, DMSO-*d_6_*) δ: 3.27-3.39 (m, 2H, CH_2_), 3.92 (s, 3H, CH_3_), 3.93 (s, 3H, CH_3_), 4.51 (t, 2H, *J* = 6.1 Hz, CH_2_), 7.00 (dd, 1H, *J* = 8.9, 2.5 Hz, H-6′), 7.26 (dd, 1H, *J* = 7.9, 4.7 Hz, H-5′′), 7.61 (d, 1H, *J* = 8.9 Hz, H-7′), 7.71 (s, 1H, H-5), 7.94 (d, 1H, *J* = 2.5 Hz, H-4′), 7.99 (bs, 1H, NH_2_), 8.17 (s, 1H, Ar), 8.19 (s, 1H, Ar), 8.39 (dd, 1H, *J* = 4.7, 1.4 Hz, H-6′′), 8.75 (dd, 1H, *J* = 7.9, 1.4 Hz, H-4′′), ^13^C NMR (50 MHz, DMSO-*d_6_*) δ: 31.2 (CH_3_), 38.6 (CH_32_), 43.6 (CH_2_), 55.3 (CH_3_), 102.4 (CH), 106.8 (CH), 109.1 (C), 110.3 (C), 111.4 (CH), 112.6 (CH), 116.0 (CH), 117.9 (C), 125.5 (C), 128.6 (CH), 129.6 (CH), 130.1 (CH), 131.5 (C), 142.3 (CH), 146.8 (C), 155.1 (C), 158.8 (C), 161.9 (C). Anal. Calcd. for: C_22_H_21_N_5_OS: C, 65.49; H, 5.25; N, 17.36. Found: C, 65.77; H, 5.17; N, 17.50. 

2-{5-Bromo-3-[4-(1*H*-pyrrolo[2,3-*b*]pyridin-3-yl)-1,3-thiazol-2-yl]-1*H*-indol-1-yl}ethanamine (**1m**) 

Orange solid; yield: 85%; mp: 180 °C; IR cm^−1^: 3609, 3558 (NH_2_), 3408 (NH); ^1^H NMR (200 MHz, DMSO-*d_6_*) δ: 3.29-3.40 (m, 2H, CH_2_), 4.55 (t, 2H, *J* = 5.6 Hz, CH_2_), 7.32 (dd, 1H, *J* = 7.9, 4.9 Hz, H-5′′), 7.51 (dd, 1H, *J* = 8.7, 1.8 Hz, H-6′), 7.72 (d, 1H, *J* = 8.7 Hz, H-7′), 7.82 (s, 1H, H-5), 7.99 (bs, 1H, NH_2_), 8.15 (d, 1H, *J* = 2.4 Hz, H-2′′), 8.33 (s, 1H, H-2′), 8.39 (dd, 1H, *J* = 4.9 ,1.7, H-6′′), 8.57 (d, 1H, *J* = 1.8 Hz, H-4′), 8.76 (dd, 1H, *J* = 7.9, 1.7 Hz, H-4′′), 12.22 (bs, 1H, NH); ^13^C NMR (50 MHz, DMSO-*d_6_*) δ: 38.5 (CH_2_), 43.7 (CH_2_), 108.2 (CH), 110.0 (C), 110.5 (C), 112.9 (CH), 114.0 (C), 116.1 (CH), 119.0 (C), 122.9 (CH), 125.2 (CH), 125.6 (CH), 126.6 (C), 131.2 (CH), 131.5 (CH), 135.4 (C), 140.4 (CH), 145.5 (C), 149.2 (C), 161.2 (C). Anal. Calcd. for: C_20_H_16_BrN_5_S: C, 54.80; H, 3.68; N, 15.98. Found: C, 54.91; H, 3.64; N, 16.10.

2-{5-Bromo-3-[4-(1-methyl-1*H*-pyrrolo[2,3-*b*]pyridin-3-yl)-1,3-thiazol-2-yl]-1*H*-indol-1-yl}ethanamine (**1n**) 

Yellow solid; yield: 60%; mp: 166 °C; IR cm^−1^: 3609, 3558 (NH_2_); ^1^H NMR (200 MHz, DMSO-*d_6_*) δ: 3.72–3.82 (m, 2H, CH_2_), 4.37 (s, 3H, CH_3_), 4.97 (t, 2H, *J* = 5.7 Hz, CH_2_), 7.70 (dd, 1H, *J* = 7.9, 4.7 Hz, H-5′′), 7.93 (dd, 1H, *J* = 8.8, 1.9 Hz, H-6′), 8.14 (d, 1H, *J* = 8.8 Hz, H-7′), 8.18 (s, 1H, H-5), 8.43 (bs, 2H, NH_2_), 8.59 (s, 1H, Ar), 8.74 (s, 1H, Ar), 8.82 (dd, 1H, *J* = 4.7, 1.4 Hz, H-6′′), 8.98 (d, 1H, *J* = 1.9 Hz, H-4′), 8.69 (dd, 1H, *J* = 7.9, 1.7 Hz, H-4′′); ^13^C NMR (50 MHz, DMSO-*d_6_*) δ: 31.1 (CH_3_), 38.5 (CH_2_), 43.7 (CH_2_), 107.4 (CH), 108.8 (C), 110.1 (C), 112.8 (CH), 114.0 (C), 116.1 (CH), 117.6 (C), 122.9 (CH), 125.2 (CH), 126.6 (C), 128.7 (CH), 129.0 (CH), 131.1 (CH), 135.4 (C), 142.6 (CH), 147.5 (C), 157.9 (C), 161.1 (C). Anal. Calcd. for: C_21_H_18_BrN_5_S: C, 55.76; H, 4.01; N, 15.48. Found: C, 55.50; H, 3.99; N, 15.45.

2-{5-Fluoro-3-[4-(1*H*-pyrrolo[2,3-*b*]pyridin-3-yl)-1,3-thiazol-2-yl]-1*H*-indol-1-yl}ethanamine (**1o**) 

Brown solid; yield: 91%; mp: 203–204 °C; IR cm^−1^: 3609, 3557 (NH_2_), 3379 (NH); ^1^H NMR (200 MHz, DMSO-*d_6_*) δ: 3.31–3.40 (m, 2H, CH_2_), 4.57 (t, 2H, *J* = 5.7 Hz, CH_2_), 7.19–7.37 (m, 2H, H-5′′ and H-6′), 7.76 (dd, 1H, *J* = 9.0, 4.4 Hz, H-7′), 7.82 (s, 1H, H-5), 8.02-8.11 (m, 3H, H-4′ and NH_2_), 8.20 (d, 1H, *J* = 2.3 Hz, H-2′′), 8.36–8.40 (m, 2H, H-2′ and H-6′′), 8.76 (d, 1H, *J* = 7.4 Hz, H-4′′), 12.26 (bs, 1H, NH); ^13^C NMR (50 MHz, DMSO-*d_6_*) δ: 38.5 (CH_2_), 43.7 (CH_2_), 105.5 (CH, *J_C6′-F_* = 23.7 Hz), 107.9 (CH), 110.5 (C), 110.9 (CH, *J_C4′-F_* = 25.3 Hz), 112.0 (C), 112.2 (CH), 116.1 (CH), 118.6 (C), 125.2 (C, *J_C3a′-F_* = 10.8 Hz), 125.4 (CH), 130.9 (CH), 131.5 (CH), 133.3 (C), 140.7 (CH), 146.0 (C), 149.2 (C), 158.3 (C, *J_C5-F_* = 235 Hz), 161.4 (C). Anal. Calcd. for: C_20_H_16_FN_5_S: C, 63.64; H, 4.27; N, 18.55. Found: C, 63.75; H, 4.23; N, 18.52.

2-{5-Fluoro-3-[4-(1-methyl-1*H*-pyrrolo[2,3-*b*]pyridin-3-yl)-1,3-thiazol-2-yl]-1*H*-indol-1-yl}ethanamine (**1p**) 

Yellow solid; yield: 70%; mp: 238–239 °C; IR cm^−1^: 3609, 3557 (NH_2_); ^1^H NMR (200 MHz, DMSO-*d_6_*) δ: 3.73–3.83 (m, 2H, CH_2_), 4.37 (s, 3H, CH_3_), 4.97 (t, 2H, *J* = 5.8 Hz, CH_2_), 7.62–7.74 (m, 2H, H-5′′ and H-6′), 8.13–8.19 (m, 3H, NH_2_ and H-7′), 8.39 (s, 1H, Ar), 8.55 (dd, 1H, J = 11.6, 2.5 Hz, H-4′), 8.65 (s, 1H, Ar), 8.74 (s, 1H, Ar), 8.81 (dd, 1H, *J* = 4.7, 1.4 Hz, H-6′′), 9.05 (dd, 1H, *J* = 8.0, 1.4 Hz, H-4′′). ^13^C NMR (50 MHz, DMSO-*d_6_*) δ: 31.2 (CH_3_), 38.5 (CH_2_), 43.7 (CH_2_), 105.7 (CH, *J_C6′-F_* = 23.7 Hz), 107.2 (CH), 111.0 (CH, *J_C4′-F_* = 25.9 Hz), 112.0 (CH, *J_C7′-F_* = 10.0 Hz), 112.3 (C), 116.2 (CH), 117.6 (C), 125.3 (C, *J_C3a′-F_* = 10.9 Hz), 129.0 (CH), 129.1 (CH), 131.5 (CH), 133.3 (C), 142.3 (CH), 147.0 (C), 149.3 (C), 158.0 (C), 158.6 (C, *J_C5-F_* = 265 Hz), 158.7 (C). Anal. Calcd. for: C_21_H_18_FN_5_S: C, 64.43; H, 4.63; N, 17.89. Found: C, 64.57; H, 4.57; N, 18.08.

### 3.2. Biology

#### 3.2.1. MICs Determination

MICs of the thiazole derivatives **1a**–**p** and **2a**–**u** towards free living form (planktonic) of three reference strains of *S. aureus* ATCC 25923 and 6538 and *P. aeruginosa* ATCC 15442, were determined by using a microdilution method as recommended by CLSI for bacteria that grow aerobically (CLSI) [43] and Tryptic Soy Broth (VWR International, Leuven, Belgium) as medium [44]. 

#### 3.2.2. Inhibition of Biofilm Formation (Crystal Violet Method). 

Bacterial strains were incubated in test tubes with Tryptic Soy Broth (TSB) (5 mL) containing 2% *w*/*v* glucose at 37 °C for 24 h. Afterwards, the bacterial suspensions were diluted to achieve a turbidity equivalent to a 0.5 McFarland standard. The diluted suspension (2.5 μL) was added to each well of a single cell culture polystyrene sterile, flat-bottom 96-well plate filled with TSB (100 μL) with 2% *w*/*v* glucose. Sub-MIC concentration values of all compounds were directly added to the wells to reach concentrations ranging from 100 to 0.1 μg/mL to assess IC_50_ values that are the concentrations at which the percentage of inhibition of biofilm formation (see below) is equal to 50%, we calculated this value by using a linear regression graph in Excel. Plates were incubated at 37 °C for 24 h. After biofilm growth, the content of each well was removed, wells were washed twice with sterile Phosphate-buffered saline (PBS) 1× and stained with 150 μL of 0.1% *w*/*v* crystal violet solution for 30 min. at room temperature. Excess solution was removed, and the plate was washed twice, by tap water. 33% *v*/*v* of acetic acid (125 μL) was added for 15 min to each stained well to solubilize the dye. Optical density (OD) was read at 540 nm using a microplate reader (Glomax Multidetection System Promega, Madison, Wisconsin, USA). The experiments were run at least in triplicates and three independent experiments were performed. [44]

The percentage of inhibition was calculated using the formula:

% of inhibition = [(OD growth control − OD sample)/OD growth control] × 100

#### 3.2.3. Antibiofilm Activity (Crystal Violet Method)

A suspension of bacteria (0.5 McFarland standard) was obtained using the procedure described above for the inhibition of biofilm formation test. 2.5 μL of suspension was added to each well of a 96-wheel plate containing TSB (100 μL) with 2% *w*/*v* glucose. After the growth of a biofilm (24 h old), the content of each well was removed, wells were washed up twice with sterile PBS and then filled with fresh TSB medium (200 μL). After that, a screening concentration of 100 μg/mL of the thiazole derivatives were added. The microtiter plate was sealed and incubated at 37 °C for further 24 h. The content of each well was removed, wells were washed up twice with sterile PBS (100 μL to each well) and the 96-wheel plate was placed at 60 °C for 1 h before staining with a 0.1% *w*/*v* crystal violet solution. After 30 min, plates were washed with tap water to remove an excess of stain. Biofilm formation was determined by solubilizing crystal violet staining in 33% *v*/*v* acetic acid (125 μL) for 15 min and measuring the absorbance at 540 nm using a microplate reader (Glomax Multidetection System Promega). To calculate the percentages of inhibition the formula above reported was used.

#### 3.2.4. Screening as Sortase A (SrtA) Inhibitors

The compounds **1a** and **1r,** selected for their good activity in inhibiting biofilm formation of *S.aureus*, were screened at a single dose of 100 µM (1% DMSO) in black 96-well plates (Greiner Bio-One, Kremsmunster, Austria). A known sortase inhibitor, 4-(hydroxymercuri)benzoic acid, was used as positive control. The inhibitory activity of the three compounds was assessed by quantifying the increase in fluorescence intensity upon cleavage of the fluorescence resonance energy transfer (FRET) substrate into two separate fragments resulting in the release of 5-Fam fluorescence, which can be monitored at excitation/emission = 490/520 nm. A commercial kit (SensoLyte^®^ 520 Sortase A Activity Assay Kit Fluorimetric, Cambridge Bioscience, Cambridge, UK) was used with slight modifications. Briefly, the reactions were performed in a volume of 100 µL containing 1× assay buffer, 2.5 µg/mL SrtA protease recombinant, 4 µM fluorescent peptide substrate, and the prescribed concentrations of the test compounds or the positive control. The peptide substrate without the recombinant SrtA was incubated in the same manner and used as a negative control. The reactions were conducted adding tested compounds and diluted enzyme solution to the microplate wells. Simultaneously setup the following control wells. Then sortase substrate solutions were added into each well. For kinetic reading, fluorescence at *Ex*/*Em* = 490/520 nm was continuously recorded every 5 min for 60 min. All fluorescence measurements are expressed in relative fluorescence units (RFU). 

## 4. Conclusions

Marine-derived compounds and their synthetic analogues that prevent the formation of biofilms without interfering with microbial viability could be advantageous when developing a new generation of anti-virulence agents counteracting antibiotic resistance.

New thiazole nortopsentin analogues were conveniently synthesized and tested as inhibitors of biofilm formation against Gram-positive and Gram-negative bacteria. Compounds **1a**–**p** and **2a**–**u** showed a good activity in inhibiting biofilm formation, in particular against Gram-positive bacteria. The inhibition of SrtA as a mechanism of action was investigated but results suggested that SrtA was not found to be involved in the inhibition of the biofilm formation of these compounds. 

Compounds **1a** and **1r** could be considered interesting lead compounds for the development of a new class of anti-biofilm agents useful in counteracting the phenomenon of the antibiotic resistance. 

## Figures and Tables

**Table 1:** New thiazole derivatives **1a**–**p**.
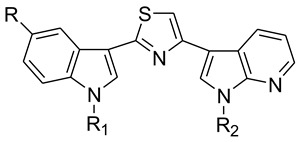

Compd.	R	R_1_	R_2_	Compd.	R	R_1_	R_2_
**1a**	H	CH_2_CH_2_NHBoc	H	**1i**	H	CH_2_CH_2_NH_2_	H
**1b**	H	CH_2_CH_2_NHBoc	Me	**1j**	H	CH_2_CH_2_NH_2_	Me
**1c**	OMe	CH_2_CH_2_NHBoc	H	**1k**	OMe	CH_2_CH_2_NH_2_	H
**1d**	OMe	CH_2_CH_2_NHBoc	Me	**1l**	OMe	CH_2_CH_2_NH_2_	Me
**1e**	Br	CH_2_CH_2_NHBoc	H	**1m**	Br	CH_2_CH_2_NH_2_	H
**1f**	Br	CH_2_CH_2_NHBoc	Me	**1n**	Br	CH_2_CH_2_NH_2_	Me
**1g**	F	CH_2_CH_2_NHBoc	H	**1o**	F	CH_2_CH_2_NH_2_	H
**1h**	F	CH_2_CH_2_NHBoc	Me	**1p**	F	CH_2_CH_2_NH_2_	Me

**Table 2:** Thiazole derivatives **2a**–**u** [39].
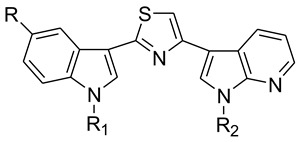

Compd.	R	R_1_	R_2_	Compd.	R	R_1_	R_2_
**2a**	H	CH_2_CH_2_OMe	H	**2l**	Br	Me	CH_2_CH_2_OMe
**2b**	H	CH_2_CH_2_OMe	Me	**2m**	F	CH_2_CH_2_OMe	H
**2c**	H	CH_2_CH_2_OMe	CH_2_CH_2_OMe	**2n**	F	CH_2_CH_2_OMe	Me
**2d**	H	Me	CH_2_CH_2_OMe	**2o**	F	CH_2_CH_2_OMe	CH_2_CH_2_OMe
**2e**	OMe	CH_2_CH_2_OMe	H	**2p**	F	Me	CH_2_CH_2_OMe
**2f**	OMe	CH_2_CH_2_OMe	Me	**2q**	F	H	CH_2_CH_2_OMe
**2g**	OMe	CH_2_CH_2_OMe	CH_2_CH_2_OMe	**2r**	H	Boc	CH_2_CH_2_OMe
**2h**	OMe	Me	CH_2_CH_2_OMe	**2s**	Br	Boc	CH_2_CH_2_OMe
**2i**	Br	CH_2_CH_2_OMe	H	**2t**	H	H	CH_2_CH_2_OMe
**2j**	Br	CH_2_CH_2_OMe	Me	**2u**	Br	H	CH_2_CH_2_OMe
**2k**	Br	CH_2_CH_2_OMe	CH_2_CH_2_OMe				

**Table 3:** Inhibition of biofilm formation, IC_50_ (µM).

Compd.	*S. aureus* ATCC 25923 µg/mL µM		*S. aureus* ATCC 6538 µg/mL µM		*P. aeruginosa* ATCC 15442 µg/mL µM	
**1a**	3.9 ± 0.2	8.4 ± 0.4	5.2 ± 0.3	11.3 ± 0.6	-	-
**1b**	13.8 ± 0.9	29.1 ± 1.9	19.3 ± 1.1	40.7 ± 2.3	15.6 ± 1.1	32.9 ± 2.3
**1c**	7.1 ± 0.1	14.5 ± 0.2	11.6 ± 0.9	23.6 ± 1.8	-	-
**1d**	14.1 ± 1.0	27.9 ± 2.0	13.1 ± 0.5	26.0 ± 1.0	-	-
**1f**	9.3 ± 0.9	16.8 ± 1.6	6.5 ± 0.5	11.7 ± 0.9	37.2 ± 2.5	67.3 ± 4.5
**1h**	36.9 ± 1.7	75.0 ± 3.4	9.3 ± 0.4	18.9 ± 0.8	13.1 ± 0.8	26.6 ± 1.6
**1i**	4.7 ± 0.3	13.0 ± 0.8	9.7 ± 0.9	26.9 ± 2.5	22.7 ± 2.1	63.1 ± 5.8
**1j**	32.9 ± 3.1	88.0 ± 8.3	6.2 ± 0.09	16.6 ± 0.2	56.1 ± 3.2	150.2 ± 8.6
**1k**	23.3 ± 1.5	59.2 ± 3.8	4.8 ± 0.1	12.3 ± 0.3	4.2 ± 0.1	10.7 ± 0.2
**1l**	48.7 ± 2.2	120.6 ± 5.4	7.2 ± 0.7	17.8 ± 1.7	24.2 ± 0.8	59.9 ± 2.0
**1m**	4.4 ± 0.1	10.0 ± 0.2	3.3 ± 0.08	7.5 ± 0.2	7.8 ± 0.09	17.7 ± 0.2
**1n**	20.1 ± 0.8	44.4 ± 1.8	5.4 ± 0.2	11.9 ± 0.4	4.6 ± 0.1	10.1 ± 0.2
**1o**	1.5 ± 0.1	3.9 ± 0.3	6.3 ± 0.4	16.6 ± 1.0	4.5 ± 0.4	11.9 ± 1.1
**1p**	0.5 ± 0.02	1.27 ± 0.05	5.2 ± 0.08	13.2 ± 0.2	3.9 ± 0.07	9.9 ± 0.2
**2d**	7.5 ± 0.2	19.3 ± 0.5	-	-	-	-
**2e**	18.6 ± 0.9	45.9 ± 2.2	25.4 ± 1.7	62.7 ± 4.2	20.5 ± 1.2	50.6 ± 3.0
**2f**	1.2 ± 0.03	2.8 ± 0.07	11.5 ± 0.7	26.7 ± 1.6	-	-
**2g**	7.9 ± 0.6	17.0 ± 1.3	11.1 ± 0.2	23.9 ± 0.4	17.7 ± 0.8	38.2 ± 1.7
**2i**	0.79 ± 0.009	1.7 ± 0.02	9.4 ± 0.3	20.7 ± 0.7	4.4 ± 0.08	9.7 ± 0.2
**2j**	0.95 ± 0.01	2.03 ± 0.02	11.2 ± 1.1	23.9 ± 2.3	19.1 ± 0.1	40.8 ± 0.2
**2k**	2.9 ± 0.02	5.6 ± 0.04	18.8 ± 1.5	36.7 ± 2.9	-	-
**2l**	2.5 ± 0.02	5.3 ± 0.04	-	-	-	-
**2m**	13.8 ± 0.7	35.1 ± 1.8	0.3 ± 0.002	0.7 ± 0.005	-	-
**2n**	0.2 ± 0.006	0.4 ± 0.012	21.0 ± 1.7	51.6 ± 4.2	-	-
**2o**	28.5 ± 1.9	63.2 ± 4.2	-	-	-	-
**2q**	13.7 ± 1.1	34.9 ± 2.8	23.1 ± 1.9	58.8 ± 4.8	-	-
**2r**	1.8 ± 0.1	3.7 ± 0.2	6.9 ± 0.1	14.5 ± 0.2	-	-
**2t**	12.9 ± 0.5	34.4 ± 1.3	7.5 ± 0.6	20.0 ± 1.6	16.3 ± 1.3	43.5 ± 3.5
**2u**	13.1 ± 0.8	28.8 ± 1.7	9.6 ± 0.9	21.1 ± 2.0	-	-
